# A Case of Acquired Hemophilia A Following SARS-CoV-2 Infection

**DOI:** 10.7759/cureus.16579

**Published:** 2021-07-23

**Authors:** Husam Hafzah, Cara McGuire, Amar Hamad

**Affiliations:** 1 Internal Medicine, Chicago Medical School at Rosalind Franklin University of Medicine and Science, North Chicago, USA; 2 Hematology and Medical Oncology, Affiliated Oncologists, Chicago Ridge, USA

**Keywords:** acquired hemophilia a (aha), hemophilia-a, sars-cov-2, factor viii inhibitor, anticoagulation

## Abstract

Acquired hemophilia A (AHA) is a rare bleeding disorder caused by antibodies against coagulation factor VIII. The majority of AHA cases are reported in an elderly population with chronic co-morbidities but can also be associated with other conditions, drugs, infections, and pregnancy. AHA is likely under-diagnosed and often unrecognized due to limited data about incidence, diagnosis, and management. We report a patient with no significant medical history who developed spontaneous ecchymoses and hematomas after a recent severe acute respiratory syndrome coronavirus 2 (SARS-CoV-2) infection complicated by a pulmonary embolism. These skin manifestations were initially thought to be related to the use of the direct oral anticoagulant apixaban, but further investigation revealed the presence of factor VIII inhibitors confirming the diagnosis of AHA. The patient was treated with prednisone and cyclophosphamide to eradicate the inhibitors with excellent response. Literature review shows a few cases of AHA after coronavirus disease 2019 (COVID-19) vaccination, H1N1 vaccination, and two cases after COVID-19 infection.

## Introduction

Acquired hemophilia A (AHA) is a rare autoimmune bleeding disorder caused by autoantibodies directed against coagulation factor VIII. Limited information on AHA epidemiology is available but it is estimated that its incidence approaches about 1.5 cases per million per year [1]. The majority of patients are elderly over the age of 50 or peri/postpartum women [2]. AHA is associated with autoimmune diseases, tumors, drugs, infections, and lymphoproliferative malignancies.

Clinical presentation in AHA is different from hereditary hemophilia A, as hereditary hemophilia A is an X-linked disorder that occurs in males at an earlier age, with positive family history and occasional hemarthroses. AHA often presents as spontaneous bleeding or hemorrhage in an otherwise healthy patient without previous personal or family history of bleeding disorders [3]. In more than 80% of cases, the hemorrhage involves the skin and presents as large hematomas or extensive ecchymoses. Bleeding can extend to muscles (intramuscular bleed), soft tissue, and mucus membranes [epistaxis, gastrointestinal (GI) bleed, hematuria] [4] but very rarely the joints. Other manifestations may include prolonged postpartum bleed and in rare cases cerebral hemorrhage. AHA bleeding can be massive, life-threatening (retroperitoneal bleed), and difficult to control leading to a relatively high mortality rate. Additionally, AHA is characterized by isolated prolonged activated partial thromboplastin time (aPTT) that is not corrected with mixing studies with evidence of autoantibodies against factor VIII. Treatment in AHA is aimed at bleeding control (if any), eradication of factor VIII inhibitors, and management of underlying conditions (if any).

Due to its rarity, the diagnosis of AHA might be overlooked. However, AHA should be suspected in any elderly patient with sudden onset of hematomas or ecchymoses without a significant inciting event or history of bleeding disorder. Further research on AHA is still needed as most of the current knowledge is drawn from anecdotal case reports and small studies. In this case, we are joining the growing number of cases that highlight potential hematologic and immunologic complications post-COVID infection. We present a case of AHA following severe acute respiratory syndrome coronavirus 2 (SARS-CoV-2) infection.

## Case presentation

A 73-year-old male with a past medical history of chronic kidney disease stage II, benign prostatic hypertrophy, and dyslipidemia was referred to the hematology clinic for evaluation. The patient had SARS-CoV-2 infection that did not require hospitalization in November of 2020. However, two weeks into the disease course, his shortness of breath became worse and he was hospitalized for hypoxia. Workup showed no signs of anemia or thrombocytopenia, normal aPTT of 32 s (normal range 24-40 s), and elevated D-dimer of 2.12 µg/mL (normal range <0.5 µg/mL) with negative bilateral lower extremity ultrasound ruling out deep vein thrombosis (DVT). A computed-tomography angiogram (CTA) of the lungs revealed scattered bilateral lower lobes lobar and segmental pulmonary emboli. The patient was started on a heparin drip bridging to direct oral factor X inhibitor apixaban and was discharged home two days later. Further laboratory workup showed negative lupus anticoagulant and antiphospholipid assays. At the time of discharge, his medications included vitamins, aspirin, apixaban, and tamsulosin. The patient had reported no side effects from anticoagulant use initially. 

**Figure 1:**
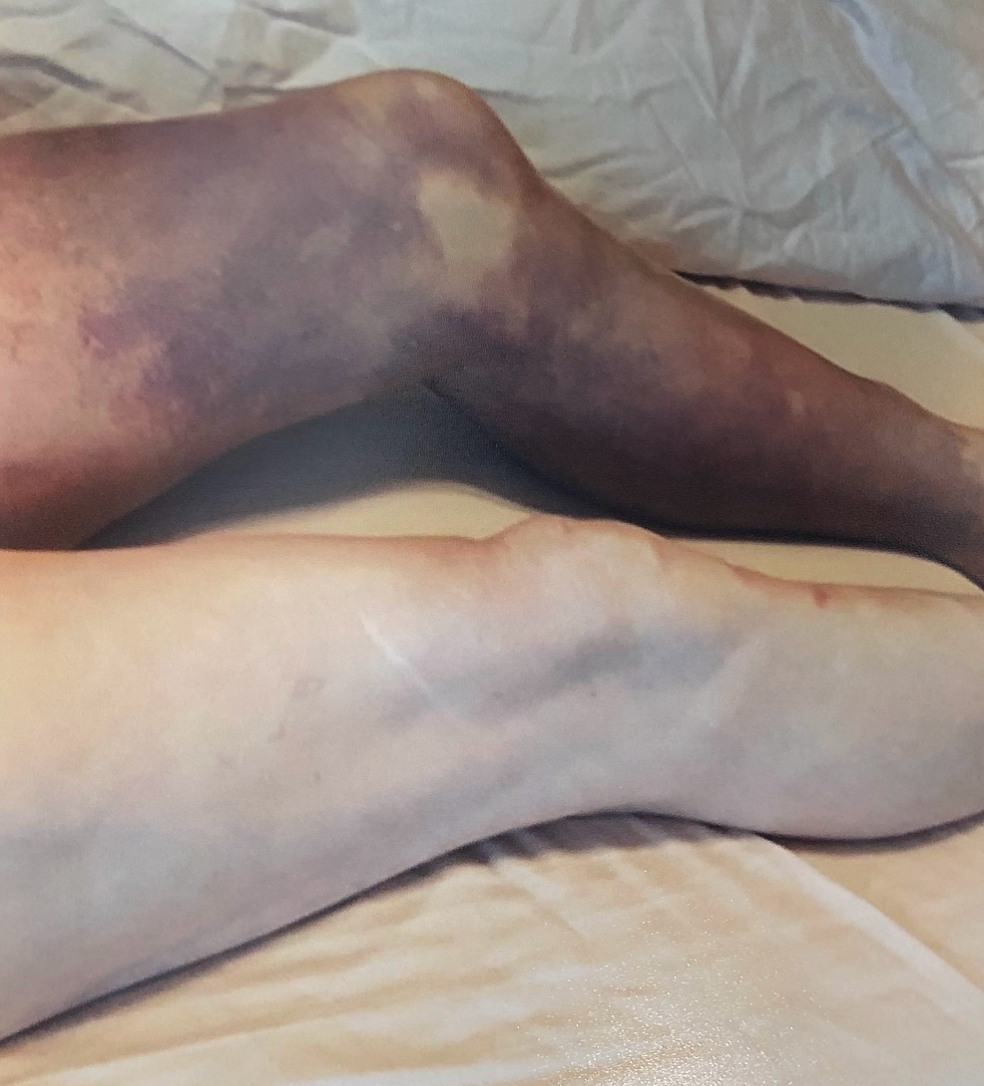
Large ecchymosis of the left thigh.

**Figure 2:**
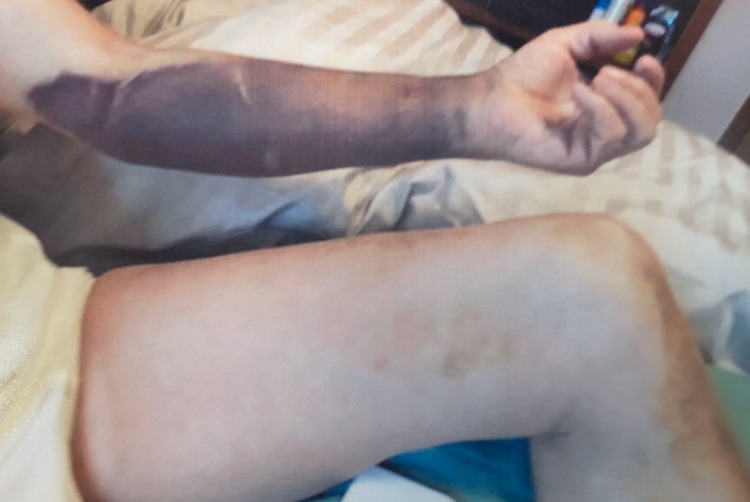
Partial resolution of left thigh ecchymosis and new ecchymosis of the left arm.

About four months later, he presented to the emergency room with complaints of a spontaneous large ecchymosis of the left thigh (Figure 1). The evaluation revealed minimal swelling of the left lower extremity that prompted a duplex ultrasound of the leg. No evidence of DVT was found and the patient was discharged home with a referral to the hematology clinic. Upon his visit to the hematology clinic, he was hemodynamically stable with partial resolution of the left thigh ecchymosis, a new large ecchymosis of the left arm (Figure 2), notable bruises to his abdomen and lower back with a spontaneous right conjunctival hemorrhage that started earlier that same day. The patient was advised to stop taking aspirin and apixaban. Laboratory studies showed INR of 1.0, aPTT of 105 s, and a CTA scan of the lungs was negative for pulmonary embolism. On his follow-up visit, the patient had a new right lower extremity ecchymosis and further testing showed a negative autoimmune panel, a negative hepatitis panel, and a negative CT scan of chest/abdomen/pelvis ruling out solid tumors. Labs showed normal factors IX and XI activity and normal von Willebrand factor antigen. A mixing study failed to correct elevated aPTT suggesting the presence of an inhibitor. Further testing showed factor VIII activity of <1% (normal range 56-141) and an extremely elevated Bethesda assay of 70.4 Bethesda units (BU). The patient was then started on prednisone and cyclophosphamide daily. Repeated labs two weeks after initiating therapy showed no signs of anemia, INR of 1.0, and aPTT of 33.2 s (normal range 24-40 s). The patient reported no medication side effects and no further bruising or hematomas. He did not endorse any new symptoms during his last visit in June 2021. He continues to be followed by hematology/oncology clinic for age-appropriate cancer screening and routine labs.

## Discussion

Acquired hemophilia A is a life-threatening, rare autoimmune bleeding disorder that typically presents in an otherwise healthy patient with spontaneous hemorrhage involving the skin, soft tissue, and mucus membranes but it can also present without any bleeding symptoms. We present a case of acquired immunohematologic complications following SARS-CoV-2 infection. while a majority of cases reviewed were related to an underlying autoimmune process, pregnancy or post-partum, malignancies and drug-related, only a limited number reported such reaction after SARS-CoV-2 infection. The exact mechanism of immunohematologic complications following SARS-CoV-2 infection is still unknown, but it is plausible that some individuals may have an antibody response that can cross-react with circulating coagulation factors and give rise to AHA and/or other complications [5].

## Conclusions

It is challenging to draw conclusions on AHA based only on a few cases or on this particular patient presentation. Further research in this domain is needed to assess any relationship between viral infections and such serious complications. However, it is important to investigate any isolated prolongation of aPTT especially after recent viral infection, immunization, or initiation of a new medication.
